# Addressing Needs of Contacts of Ebola Patients During an Investigation of an Ebola Cluster in the United States — Dallas, Texas, 2014

**Published:** 2015-02-13

**Authors:** Charnetta L. Smith, Sonya M. Hughes, Mateusz P. Karwowski, Michelle S. Chevalier, Emily Hall, Sibeso N. Joyner, Julia Ritch, Jessica C. Smith, Lauren M. Weil, Wendy M. Chung, Stephanie Schrag, Scott Santibañez

**Affiliations:** 1 Epidemic Intelligence Service, CDC; 2 Division of STD Prevention, National Center for HIV/AIDS, Viral Hepatitis, STD, and TB Prevention, CDC; 3 CDC Dallas Ebola Investigation Team; 4 Dallas County Health and Human Services

The first imported case of Ebola virus disease (Ebola) diagnosed in the United States was confirmed on September 30, 2014; two health care workers who cared for this patient subsequently developed Ebola (1). Since then, local, state, and federal health officials have continued to prepare for future imported cases, including developing strategies to identify and monitor persons who have had contact with an Ebola patient. This report describes some of the needs of persons who were contacts of Ebola patients in Texas. It is based on requests received from contacts in the course of daily contact tracing interactions and on how those needs were met through community partnerships. Meeting the needs of contacts of the Ebola patients was essential to successful contact tracing, which is critical to interrupting transmission. Although a formal needs assessment of contacts was not conducted, this report provides important information for preparing for an importation of Ebola. Anticipating the nonclinical needs of persons under public health surveillance includes addressing potential concerns about housing, transportation, education, employment, food, and other household needs. Ensuring necessary supports are in place for persons who are asked to refrain from entering public venues can impact their willingness to comply with voluntary and mandated quarantine orders. Engagement with a wide range of community partners, including businesses, schools, charitable foundations, community and faith-based organizations, and mental health resources would enhance public health emergency preparedness for Ebola by readying resources to meet these potential needs.

A total of 179 contacts (including the two health care workers who became infected and whose illnesses subsequently were counted as cases) of the three patients with Ebola diagnosed in Texas were identified, including 149 health care workers, 20 community contacts, and 10 persons who had been transported in the same ambulance that transported the first patient with Ebola before it was completely cleaned and disinfected (1). The 20 community and 10 ambulance contacts included the following at-risk or vulnerable populations (2): school-aged children (eight), non-English speakers (Spanish, Armenian, and Nepali) (three), persons with complex chronic medical conditions (two), and persons experiencing homelessness (one). The person experiencing homelessness was initially difficult to locate. This person was given temporary quarters and quarantined to facilitate compliance with monitoring. Contact tracers from local and state health departments and CDC actively monitored contacts through twice-daily symptom and temperature checks at least 6 hours apart, once by telephone and once in-person (3). Five of the community contacts and two ambulance contacts were isolated under legal control orders, and at least 20 health care worker contacts voluntarily self-quarantined. A total of 68 health care worker contacts were eventually placed under controlled movement restrictions directing avoidance of public congregate settings, such as grocery stores and restaurants, as well as avoidance of long distance travel by commercial conveyances (2). Contacts often reported their needs and experiences to contact tracers on an ad hoc basis, including their feelings of social isolation. Specific needs were often related to the degree of social isolation experienced by the contacts. Some contacts reported difficulty obtaining basic necessities such as food, diapers, medical supplies, and refills of prescription medications. The 20 community contacts were part of seven households and included eight working adults, all of whom were excluded from work by employers. Six out of seven households required either financial support for rent and utilities and/or other assistance in procuring basic necessities such as food. Two households of the community contacts stated that they felt unsafe leaving their homes because of stigmatization by others in their community after their photos, names, and addresses had been published in the media.

All eight contacts who were children were excluded from school or daycare during the duration of the 21-day monitoring period. Procurement of childcare was a challenge encountered by families who were requested by schools or daycares to keep children home because of concerns that their children posed a risk to others in the school. Continuity of children’s education was especially challenging in families without access to technology for home study. Witnessing the first Ebola patient’s health deteriorate, and subsequently learning that two health care workers were ill, further heightened anxiety among health care contacts. More than three quarters of community and health care worker contacts reported stress, social isolation, or stigma. A common report among health care worker contacts was that caring for the index patient was emotionally taxing. The majority of the health care worker contacts experienced some degree of anxiety about possibly becoming ill or infecting their family members.

## Discussion

By working with local and charitable organizations, the contact tracing team was able to link contacts to sources of financial aid. Even among those who did not require financial assistance, seven (3.9%) requested help changing pre-existing reservations for airline flights scheduled for their monitoring period so that they could comply with their movement restrictions. Contact tracers also found that recognizing unique cultural, linguistic, and socioeconomic differences helped ensure contacts’ compliance with monitoring, particularly among the community contacts (4). For example, the first Ebola patient was Liberian, and many of his contacts were part of the local Liberian community. Relationships between the contacts and the contact tracing team were strengthened when the team worked with aid organizations to provide familiar food and clothing in a culturally sensitive manner. The contact tracing team worked with local school districts and charitable foundations to provide laptops, textbooks, and school supplies to ensure students could access course materials. Teachers designed lesson plans and assignments that could be completed at home. Physicians from the Dallas County Medical Society also volunteered to present current information about Ebola to school administrators, teachers, and parents to help minimize stigma and ensure that all students would be welcomed back into their schools. Contact tracers also served as an important source of emotional support. In addition, social workers volunteered their time to provide counseling services to contacts, although only one contact used these services.

The findings in this report are subject to at least one limitation. This assessment did not include a formal, structured survey to quantify needs and thus was limited to ad hoc contact tracing data collected over the course of the public health response. Preparedness for similar responses in the future would benefit from developing a simple database to quantify and track contact needs and align them with community partner resources, including social workers in order to better address these issues.

What is already known on this topic?

Little has been reported on the implications of being identified as an Ebola contact or on the nonclinical needs that might arise for this population.

What is added by this report?

Contact tracers from Dallas County Health and Human Services, the Texas Department of State Health Services, and CDC actively monitored 179 contacts of three Ebola patients in Texas, including 149 health care workers, 20 community contacts, and 10 persons who had been transported in the ambulance that transported the first patient with Ebola. Contacts were monitored daily with symptom and temperature checks. All contacts experienced some type of movement restriction. Meeting the needs of contacts of Ebola patients, including basic needs for food, financial assistance, and education, was essential to successful contact tracing, which is critical to interrupting transmission.

What are the implications for public health practice?

Engagement with a wide range of community partners, including businesses, schools, charitable foundations, community and faith-based organizations, and mental health resources would enhance public health emergency preparedness for Ebola. When this is done before the identification of an Ebola case, it can provide a useful basis for addressing the needs of persons identified as contacts of an Ebola case, and facilitates successful contact tracing during an Ebola investigation.

Contact tracing in this challenging setting involved more than monitoring temperatures and checking for symptoms. Meeting the needs of contacts was essential to effective contact tracing and therefore was critical to interrupting Ebola transmission in Dallas. Although this report focuses on preparing for possible future cases in the United States, lessons learned from this contact tracing experience might be useful in other sites where there are cases of Ebola. Unless preparations are made to address the needs of Ebola contacts, responders might have difficulty following all possible contacts and as a result, contact tracing might be incomplete. Partnering with businesses, schools, charitable foundations, community and faith-based organizations, and mental health resources before an Ebola case is identified is an important part of public health emergency preparedness and will be useful for responding to possible future cases of Ebola.
